# Acceptability of a Novel Prehospital Emergency Care System Evaluation Tool Among Southeast Asian Stakeholders: A Cross-Sectional Survey

**DOI:** 10.5811/westjem.61956

**Published:** 2026-11-01

**Authors:** John T. Bokman, Ali Haedar, Anjni Joiner, Lauren Hart, Sarah Binti Shaikh Abdul Karim, Faith Joan Mesa-Gaerlan, Pin Pin Pek, Sattha Riyapan, Marcus EH Ong, Audrey L. Blewer, Gayathri Devi Nadarajan

**Affiliations:** * Duke University School of Medicine, Durham, North Carolina; † Saiful Anwar General Hospital, Faculty of Medicine, Universitas Brawijaya, Indonesia; ‡ Duke University, Department of Emergency Medicine, Durham, North Carolina; § Health Services Research and Population Health, Duke-NUS, Singapore, Singapore; ¶ University of Philippines, Philippine General Hospital and Southern Philippines Medical Center, Davao, Philippines; || Duke University School of Medicine, Department of Family Medicine and Community Health and Department of Population Health Sciences, Durham, North Carolina; # International Medical School, Management and Science University, Shah Alam, Malaysia; ** Mahidol University, Siriraj Hospital, Faculty of Medicine, Thailand; †† Singapore General Hospital, Department of Emergency Medicine, Singapore

## Abstract

**Introduction:**

Prehospital emergency care (PEC) systems vary widely in structure, capacity, and stage of development, and existing evaluation tools are not tailored to the contextual needs of Southeast Asia. To address this gap, the Prehospital Emergency Care System Evaluation Tool (PECSET) was developed as a 32-element, consensus-derived framework to support system assessment, prioritization, and quality improvement. The objective of this study was to evaluate the prospective acceptability of the PECSET framework among Southeast Asian prehospital stakeholders using the theoretical framework of acceptability (TFA) and to identify regional variation in implementation priorities.

**Methods:**

We conducted a cross-sectional survey of PEC professionals from the Philippines, Thailand, Vietnam, Indonesia, and Malaysia between April 2023 and June 2024. Participants were recruited from six resuscitation training conferences and attended a standardized two-hour educational session before completing an anonymous survey mapped to TFA constructs. We assessed differences between countries using Pearson chi-square tests. Our primary outcome measure was acceptability scores across five TFA domains (affective attitude, intervention coherence, opportunity costs, self-efficacy, and perceived effectiveness). Secondary outcomes included identification of high-priority implementation elements.

**Results:**

Participants (*N* = 274; 66% response rate) included physicians (46%, *n* = 126), prehospital professionals (30%, *n* = 82), and medical directors (15%, *n* = 41). Intervention coherence was high: 92% to 100% of participants were likely or extremely likely to use PECSET for quality improvement. However, geographic variation existed; a small subset in Indonesia and Vietnam (*n* = 6) reported they were “unlikely” to use the tool, compared to none in Thailand, Malaysia, and the Philippines (*P* = .04). Regarding self-efficacy, while 100% (*n* = 274; 95% CI, 98–100%) reported confidence in application, only 20% (*n* = 55; 95% CI,15–25%) were “extremely confident.” Affective attitude was positive, with 97% (*n* = 266) intending to integrate the tool; only Vietnam (*n* = 4; 5% [95% CI, 2–10%]) and Indonesia (*n* = 2; 2% [95% CI, 0–13%]) indicated hesitation (*P =* .24). Secondary outcomes identified “layperson public awareness and education programs” as the top implementation priority across all five nations, although priorities varied.

**Conclusion:**

The Prehospital Emergency Care System Evaluation Tool demonstrated high acceptability among potential end-users across Southeast Asia, indicating readiness for broader implementation and future evaluation of feasibility, fidelity, and system-level impact. As a stakeholder-informed evaluation framework, PECSET offers a practical approach to strengthening PEC systems in Southeast Asia and beyond.

## INTRODUCTION

A quality prehospital emergency care (PEC) system improves healthcare accessibility and ensures timely care for patients suffering from high-acuity conditions. Its importance is particularly evident in resource-limited settings, where 54% of mortality can potentially be averted with improved emergency care.^1^–^3^ However, patients in these settings are more likely to face delayed entry into the emergency care system due to unreliable transportation, poor road quality, and greater physical distance to definitive care.^4^,^5^ These barriers provide opportunities for a PEC system to significantly improve patient outcomes by increasing access, use, and effectiveness of the healthcare system.^6^–^9^

Despite the potential impact of establishing robust PEC systems, these systems are typically poorly funded and deprioritized at the local and national level in many resource-limited settings.^10^–^16^ Existing PEC systems in low-resource areas lack properly trained personnel, vital medical equipment, and adequate transport capability.^17^ One of the most significant steps in improving systems in these settings is a clear set of standards to guide their development.^18^,^19^

While several emergency care assessment frameworks exist, including the World Health Organization Prehospital Emergency Care Assessment Tool (PEAT) and tools developed by Howard et al and Mehmood et al,^20^–^22^ these instruments were primarily designed to support nascent PEC systems with broad global applicability and may not fully capture the needs of PEC systems in Southeast Asia. Many PEC systems in Southeast Asia are better characterized as being in an “intermediate” stage of development, with foundational structures in place but substantial heterogeneity in capacity, scope, and performance.^23^–^26^ This context highlights the need for an evaluation tool that is regionally tailored to Southeast Asia and sufficiently flexible to account for variable system maturity.

To address this gap, the Prehospital Emergency Care System Evaluation Tool (PECSET) was developed.^27^ The PECSET differs from existing frameworks in several key ways: It is specifically designed for Southeast Asian systems at an early developmental stage; it incorporates a prioritization logic that accounts for implementation status, feasibility, and relative importance; and it was co-developed with intended users in an effort to enhance content validity and contextual relevance. As a novel system-level intervention, understanding PECSET’s acceptability among potential users is a critical prerequisite for adoption and scale-up.^28^ Therefore, the primary objective of this study was to evaluate the acceptability of PECSET among individuals working within Southeast Asian PEC systems, mapped to domains within the theoretical framework of acceptability (TFA). 28 Secondary exploratory outcomes included participants’ intentions to use and share the tool, as well as descriptive assessments of current implementation status and prioritization of PECSET elements within their systems.

**Population Health Research Capsule**

What do we already know about this issue?

*Existing prehospital emergency care evaluation tools are not well suited to Southeast Asia.*

What was the research question?

*Is the Prehospital Emergency Care System Evaluation Tool (PECSET) acceptable to Southeast Asian prehospital emergency care stakeholders?*

What was the major finding of the study?

*100% reported confidence applying PECSET (95% CI, 98–100%); 97% intended to use it (95% CI, 94–99%).*

How does this improve population health?

*An acceptable evaluation tool can help prioritize interventions, guide quality improvement, and strengthen prehospital emergency care systems across Southeast Asia.*

## METHODS

### Study Design and Setting

This cross-sectional study assessed the acceptability and perceived utility of PECSET among PEC system professionals in Southeast Asia. Data were collected between April 26, 2023, and June 7, 2024, across five countries (Philippines, Thailand, Vietnam, Indonesia, Malaysia). The study was reviewed and deemed exempt by the National University of Singapore Institutional Review Board (Ethics Number: NUS-IRB-2021-136).

### Development of the Prehospital Emergency Care System Evaluation Tool

The PECSET was developed through a four-stage modified Delphi consensus process involving 32 prehospital emergency care experts from 12 Asian countries.^27^ Initially, 32 potential system elements were identified via a scoping review, focus group discussions, and surveys. In the first two Delphi rounds, experts evaluated these elements based on their importance and feasibility in low-resource settings. The final two rounds focused on prioritizing “core” elements essential for systems in early development. While the process identified 12 core building blocks, all 32 elements were retained to provide a tiered evaluation approach suitable for early, intermediate, and advanced PEC systems (Table 1).

### Participants

Participants were PEC professionals attending a Resuscitation Academy (RA) (https://www.resuscitationacademy.org/), a structured two-day training program designed to improve cardiac arrest survival outcomes. Participants were recruited from six RAs: Clark, Philippines (April 2023); Lombok, Indonesia (July 2023); Bangkok, Thailand (December 2023); Ho Chi Minh, Vietnam (January 2024); Danang, Vietnam (February 2024); and Selangor, Malaysia (June 2024). All attendees had the opportunity to participate in the study. By targeting individuals with active roles in PEC system operations and quality improvement initiatives, the study ensured that respondents had relevant expertise to evaluate the acceptability and feasibility of PECSET. A two-hour session on PECSET was incorporated into the RA, during which participants learned about the development, purpose, and elements of the tool. After the session, participants were invited to provide feedback on PECSET via an anonymous electronic survey accessed through a QR code or link. Consent was obtained electronically. Only one response per participant was allowed.

### Survey Instrument

The survey focused on assessing acceptability, feasibility, and perceived utility of PECSET. It included two components:

1. PECSET Implementation: Participants completed PECSET for their PEC system, indicating the extent to which PECSET core elements were implemented, partially implemented, or not implemented. (Appendix 1).^27^
2. Feedback on Acceptability: Participants reported demographics and responded to Likert-scale items evaluating their confidence in using PECSET, intention to apply the tool, perceived effectiveness, and anticipated impact on patient care and system outcomes. They then prioritized two specific PECSET elements to focus on over the next six months (Appendix 2).

### Survey Development

The feedback portion of the survey was designed to systematically capture participant perceptions of the PECSET tool. It was drafted to cover desired domains for understanding acceptability and feasibility of the tool (respondent demographics, confidence, intention for use). A preliminary version of the survey was evaluated by PEC experts and prospective end users from across Southeast Asia (including PEC researchers, international fellows, and system leaders), after which revisions were made to improve content and flow based on their input. This process ensured the survey addressed the specific needs and contexts of PEC systems in resource-limited settings in the region.

### Validity Framework

Content validity of the survey instrument was established through review by both PEC experts and potential end users from across Southeast Asia. To support construct validity and enable a systematic assessment of acceptability, survey items were mapped to the TFA.^28^ For example, questions assessing participants’ confidence in applying PECSET mapped to self-efficacy, while items on anticipated impact of PECSET aligned with perceived effectiveness. Likelihood of use and sharing the tool captured aspects of intervention coherence and affective attitude (Table 2). By grounding the survey in TFA, the study ensured a structured, theory-driven assessment of acceptability across key domains relevant to PECSET.

### Data Collection and Analysis

We collected and managed data using REDCap, version 13.7 (Vanderbilt University). Categorical variables were reported as frequencies and percentages with corresponding 95% confidence intervals for key acceptability outcomes. We evaluated associations between categorical variables using the Pearson chi-square test of independence.

For implementation status, respondents could choose from the following four options: (1) fully implemented; (2) partially implemented; (3) not implemented; or (4) unsure. To calculate a single implementation score per element, per country, a value of 1 was assigned to responses of fully implemented, a value of 0.5 to responses of partially implemented, and a value of 0 to responses of not implemented. For the purposes of this score, responses of unsure were removed. We calculated the total value of responses based on the values assigned above and then divided by the total number of responses to calculate an average implementation score. Optional free-text comments were collected following Likert scale questions to allow participants to provide additional feedback. These responses were reviewed but were not included in formal qualitative analysis because the comments were generally brief, heterogeneous, and submitted in multiple languages. Consistent with the concept of information power, the free-text responses were determined to lack sufficient depth and informational content to support qualitative analysis.^29^

## RESULTS

### Participant Characteristics

The study involved 274 participants from five Southeast Asian countries (Indonesia, Thailand, the Philippines, Vietnam, and Malaysia), a 66% response rate of RA attendees (Table 3). No participants were excluded from analysis. Respondents represented a diverse range of PEC professionals including 46% physicians, 30% prehospital professionals (paramedics, emergency medical technicians [EMT], prehospital nurses), 15% medical directors, 14% hospital nurses, 3% administrators, and 8% dispatchers/telecommunicators. Participants originated from a relatively even distribution of PEC urbanicity with 31% working in mixed urban-rural settings, 39% in rural, and 30% in urban settings. Countries such as Indonesia and Vietnam had a higher proportion of rural participants (73%), while Thailand and the Philippines had more urban-based emergency medical services (EMS) professionals (51% and 39%, respectively). Participants were relatively new to working in the PEC setting, with 56% of participants reporting 0–5 years of experience.

### Intervention Coherence and Intention to Use

Likelihood of using PECSET for quality improvement was used as an indicator of intervention coherence and intended adoption. The majority of participants expressed that they were likely or extremely likely to use the elements for quality improvement with 93% (*n* = 38; 95% CI, 80–98%) from Indonesia, 100% (*n* = 28; 95% CI, 88–100%) from Thailand, 99% (*n* = 132; 95% CI, 95–100%) from Vietnam, 100% (*n* = 35; 89–100%) from the Philippines, and 100% (*n* = 36; 95% CI, 90–100%) from Malaysia (Figure 1). Indonesia and Vietnam had a small but significant percentage who reported that they were somewhat unlikely or extremely unlikely to use PECSET for quality improvement (*P* = .04).

### Self-Efficacy and Perceived Effectiveness

All participants reported confidence in applying PECSET within their local PEC systems with no significant difference between countries (*P* = .32). However, only 20% (*n* = 55; 95% CI, 15–25%) were extremely confident that they could apply PECSET within their local PEC. Participants were also optimistic that PECSET would improve care outcomes in their regions with 95% (*n* = 259; 95% CI, 91–97%) of all participants indicating that they were very or extremely confident with PECSET.

### Affective Attitude and Dissemination Intent

Participant plans to integrate PECSET into their local systems reflect affective attitude toward the tool. Most participants indicated plans to integrate PECSET into their PEC systems, with 97% (*n* = 266; 95% CI, 94–99%) of all respondents stating their intention to use the tool. Only participants from Vietnam and Indonesia indicated hesitation with 5% (*n* = 4; 95% CI, 2–10%) and 2% (*n* = 1; 95% CI, 0–13%), respectively, responding that they did not plan to use PECSET. However, the observed hesitancy was not significant from the other countries (*P* = .24). Nearly all participants showed a high likelihood of sharing the tool with other systems or medical directors (99% [*n* = 272; 95% CI, 97–100%]). Only 1% (*n* = 1; 95% CI, 0–4%) from Vietnam and 2% (*n* = 1; 95% CI, 0–13%) from Indonesia stated that they were extremely unlikely to share this tool, but these responses did not differ significantly from respondents from other countries (*P* = .29).

### Prioritization of Prehospital Emergency Care System Evaluation Tool Elements

Participants were also asked to indicate which elements they would prioritize implementing in their healthcare system if they were to use PECSET. The top two elements chosen by members of each country are displayed in Table 4. Some countries, such as Indonesia and Vietnam, focused on improving infrastructure and capacity. Others such as Thailand and Malaysia emphasized protocols, training, and quality assurance to improve PEC outcomes. All countries chose “layperson public awareness and education programs” as a top element on which to focus. Figure 2 shows the current implementation status of the core elements for each country as assessed by the participants. Aggregate scores were given to the elements based on their implementation status with higher scores indicating a higher implementation status.

## DISCUSSION

Implementation science research in PEC remains relatively limited, with a recent systematic review identifying a paucity of studies evaluating implementation strategies and outcomes in EMS settings.^30^ While no prior studies have assessed the acceptability of a comprehensive prehospital evaluation framework, Pap et al evaluated the acceptability of a suite of prehospital quality indicators among Australian EMS clinicians.^31^ Similar to our findings, they reported high overall acceptability of the proposed indicators, attributing this to their evidence-informed development, clarity, practicality, and perceived clinical relevance. Unlike Pap et al, whose work focused on refining individual quality indicators by evaluating their characteristics, our study assessed implementation-related perceptions of an integrated PEC system evaluation framework, demonstrating early implementation readiness through high levels of self-efficacy, intervention coherence, perceived effectiveness, and intention to use.

### Acceptability and Related Implementation Outcomes

Despite representing a wide range of PEC roles and system types, participants consistently reported high confidence in their ability to use PECSET, which reflects strong self-efficacy, a core construct within the TFA.^28^ Participants also believed the tool could improve patient care and system performance, indicating high perceived effectiveness. In addition, respondents’ plans to apply and share PECSET demonstrate strong intervention coherence, reflecting clear understanding of the tool’s purpose, structure, and relevance within their local contexts. Together, these findings indicate that PECSET is highly acceptable to its intended users and is well positioned for further implementation testing.

Although the survey was designed to assess acceptability, several items align with other constructs from Proctor’s implementation outcomes framework.^32^ Participant confidence in applying PECSET suggests early perceptions of feasibility, while stated intentions to use and disseminate the tool point toward spread potential. Beliefs that PECSET would improve patient care reflect perceived appropriateness, meaning users viewed the tool as a good fit for their systems. These findings should be interpreted cautiously, but they highlight areas for future studies to explore using dedicated measures of feasibility, adoption, fidelity, and system-level impact.

### Diversity and Generalizability

The study included a multinational sample representing a wide range of PEC system types, including varying levels of urbanization, system development, and operational priorities. Participants encompassed medical directors, EMS clinicians, dispatchers, nurses, and administrators, roles that collectively span the spectrum of PEC responsibilities. This diversity strengthens confidence that the findings reflect perspectives of the broader population of potential PECSET users and enhances external validity, suggesting that the tool may be generalizable and adaptable to multiple PEC settings throughout Southeast Asia. While most respondents had relatively limited PEC experience (≤ 5 years), their consistent responses across countries and roles indicate that PECSET is understandable and acceptable even to less-experienced end-users.

### Perceived Clinical and Operational Value

High participant-reported confidence in using PECSET and in its potential impact on patient outcomes is critical to successful adoption. Although no known prior studies have evaluated patient outcomes directly before and after implementing a PEC evaluation tool, the core elements of PECSET (eg, dispatch-assisted instructions, quality assurance programs, universal emergency numbers, layperson education) are individually supported by evidence of morbidity and mortality benefit.^33^–^35^ These alignments reinforce the perceived clinical value expressed by respondents and support the tool’s potential to guide meaningful system enhancements.

### Adoption Potential and Implementation Readiness

Acceptability must be translated into intent to use for implementation to progress. Most respondents indicated concrete plans to apply PECSET in their systems and to share it with colleagues, reflecting strong perceived usefulness and alignment with real-world PEC needs in Southeast Asia. The high levels of confidence reported by participants also suggest that the RA training sessions effectively communicated the tool’s purpose and applications. This foundation provides a favorable environment for future training and dissemination.

### Adaptability to Local System Priorities

Differences in the PECSET elements prioritized by respondents highlight the heterogeneity of PEC challenges across Southeast Asia. These variations underscore the importance of a tool that is structured yet flexible, allowing local stakeholders to tailor evaluations to system-specific needs. Such adaptability enhances PECSET’s relevance across settings and offers direction for targeted resource allocation, quality improvement initiatives, and capacity-building efforts.

### Potential Impact and Framework for Developing Region-Specific Tools

The high acceptability of PECSET across a diverse, multinational sample suggests it has the potential to improve system-level performance in Southeast Asian PEC systems. This tool provides a structured, evidence-informed framework to evaluate and prioritize prehospital care interventions, guiding resource allocation, quality improvement, and policy decisions across varied contexts. Its development, incorporating stakeholder engagement, iterative refinement through Delphi consensus, and TFA-based survey design to demonstrate acceptability, offers a model for creating other region-specific evaluation tools. The principles of stakeholder engagement, adaptability, and focus on actionable elements can inform other tools addressing regional challenges in prehospital care or health system evaluation. These findings also lay the groundwork for future studies assessing PECSET’s impact on measurable system outcomes.

## LIMITATIONS

Several limitations should be acknowledged. Only individuals who attended the Resuscitation Academy were approached for participation, introducing potential sampling bias. These individuals are likely more motivated, reform-oriented, and engaged in quality improvement than the broader EMS workforce, which may limit generalizability and inflate estimates of acceptability and intention to use. As early adopters are the intended target audience for initial dissemination, their perspectives remain highly relevant; however, EMS personnel who are unfamiliar with the framework or who have not participated in similar educational initiatives may be less inclined to adopt or implement it in practice.

Social desirability bias may also have influenced responses, as the survey was administered immediately following a training session, potentially leading to more favorable feedback. Anonymity was maintained to mitigate this effect. The timing of outcome collection may also have introduced recency bias and resulted in more favorable ratings. In addition, perceptions were assessed at a single time point, and no follow-up survey was conducted. Consequently, the study cannot determine whether these positive perceptions were sustained over time or with memory decay. Nonresponse bias is also possible given the 34% nonresponse rate.

The survey relied primarily on fixed-choice items, limiting the ability to capture nuanced perspectives or contextual barriers and facilitators influencing acceptability. Future studies should incorporate qualitative or mixed-methods approaches, such as interviews or focus groups, to provide deeper insight into user experiences.

Formal psychometric evaluation of the survey instrument was beyond the scope of this study. Construct validity and reliability were not formally assessed, and internal consistency measures were not calculated, as the survey was not designed to measure multiitem latent constructs. Although items were informed by the theoretical framework of acceptability to support conceptual alignment, findings should be interpreted as an initial descriptive assessment rather than a validated measurement instrument. Future work should include formal reliability testing and construct validation.

Finally, the survey was administered only in English and Vietnamese, neither of which was the first language for many participants and may have influenced interpretation despite expert review for clarity.

## CONCLUSION

We demonstrated that PECSET is acceptable to potential en users across diverse prehospital systems in Southeast Asia. Participants expressed confidence in using the tool, recognized its relevance to their practice, and believed it could improve patient care, indicating self-efficacy, perceived effectiveness, and intervention coherence. These early indicators of acceptability, combined with consistent intentions to use and disseminate PECSET, suggest that the tool is well positioned for broader implementation. As a stakeholder-informed evaluation tool, PECSET offers a practical framework for strengthening prehospital emergency care systems in Southeast Asia and has the potential to be adapted for other settings. Future work should focus on assessing feasibility, fidelity, and system-level outcomes as PECSET is introduced into real-world practice.

## Supplementary Information





## Figures and Tables

**Figure 1:**
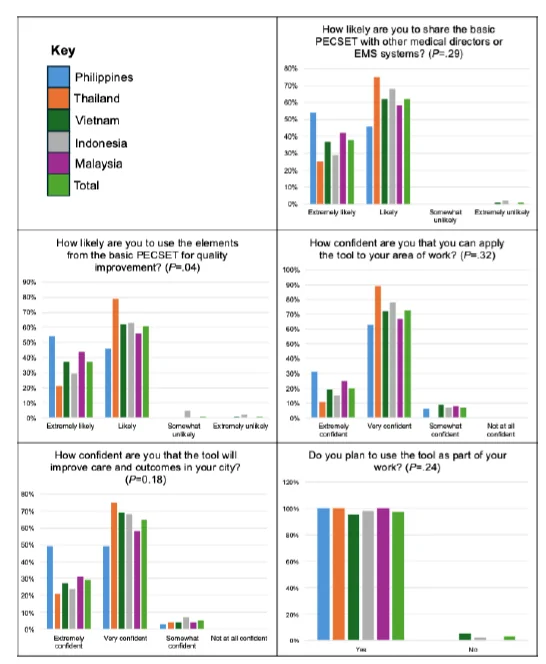
Series of bar charts illustrating the distribution of participant responses regarding their likelihood to share and use the tool, as well as their confidence in applying it and its potential to improve local care outcomes. *PECSET*, Prehospital Emergency Care System Evaluation Tool.

**Figure 2:**
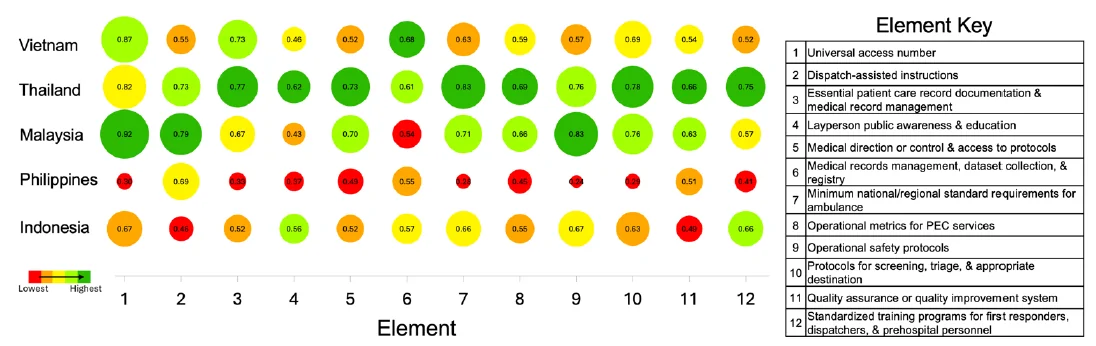
Color-coded scale (red to green) and numerical aggregate scores showing the current implementation level of 12 core system elements, as reported by participants the five participating countries, in a study of the acceptability of the PECSET among Southeast Asian stakeholders. *PECSET*, Prehospital Emergency Care System Evaluation Tool.

**Table 1:** The 32 elements of the Prehospital Emergency Care System Evaluation Tool framework, in a study of the acceptability of this tool among Southeast Asian stakeholders.

	PECSET Core Elements
1	Universal access number for all medical emergencies
2	Dispatch-assisted instructions (eg, first aid, CPR instructions)
3	Essential patient care record documentation and medical record management
4	Layperson public awareness and education programs
5	Medical direction or control and access to protocols
6	Medical records management, dataset collection, and registry
7	Minimum national or regional standard requirements for an ambulance
8	Operational metrics for prehospital emergency care services
9	Operational safety protocols
10	Protocols for screening, triage and appropriate destination
11	Quality assurance or quality improvement system in place
12	Standardized training programs for community first responders, dispatchers, and prehospital personnel

	Non-Core Elements

13	Ability to receive feedback from patients using the ambulance services
14	Air transport infrastructure
15	Dedicated medical teams in hospital to receive prehospital patients
16	Feedback mechanism for prehospital staff
17	Formal interfacility transfer system with protocols and monitoring/quality assurance
18	Formal recognition of prehospital care within a governmental structure
19	Infrastructure for the development of teleconsult or teleradiology service
20	Mechanism to obtain hospital or community data on patient outcomes
21	Networks with representatives spanning across the chain of survival
22	Prehospital notification and/or activation system
23	Prehospital staff wellness program
24	Protocols for Infection control/disease outbreak
25	Protocols for mass casualty incidents and disaster management
26	Protocols for prehospital surge capacity situations
27	Protocols to respond to surge in the ED
28	Providing appropriate care bundle on the ambulance
29	Public AED availability and usage
30	Ratio of numbers of prehospital ambulances and staff to the population
31	Scope of practice defined at a national or regional level
32	Standardized prehospital clinical assessment/ scoring tool

*AED*, automated external defibrillator; *CPR*, cardiopulmonary resuscitation; *ED*, emergency department; *PECSET*, Prehospital Emergency Care System Evaluation Tool.

**Table 2:** Mapping of survey questions to the theoretical framework of acceptability in a study of the acceptability of the Prehospital Emergency Care System Evaluation Tool among Southeast Asian stakeholders.

Survey Questions	Mapped TFA Component(s)
How likely are you to use the elements from the basic PECSET for quality improvement?	Affective attitude; intervention coherence, opportunity costs
How likely are you to share the basic PECSET with other medical directors or EMS Systems?	Affective attitude; intervention coherence
How confident are you that you can apply the tool to your area of work?	Self-efficacy
How confident are you that the tool will improve care and outcomes in your city?	Perceived effectiveness

*EMS*, emergency medical services; *PECSET*, Prehospital Emergency Care System Evaluation Tool; *TFA*, theoretical framework of acceptability.

**Table 3:** Participant characteristics in a study of the acceptability of the Prehospital Emergency Care System Evaluation Tool among Southeast Asian stakeholders.

Characteristic	Philippines	Thailand	Vietnam	Indonesia	Malaysia	Total
Number of respondents (response rate)	35 (71%)	28 (68%)	134 (57%)	41 (80%)	36 (88%)	274 (66%)
Credentials*						
MD/MBBS/DO	7 (20%)	20 (71%)	93 (69%)	14 (34%)	10 (28%)	144 (53%)
RN/MSN	19 (54%)	6 (21%)	35 (36%)	20 (49%)	0 (0%)	80 (29%)
EMT/Paramedic	12 (34%)	2 (7%)	2 (1%)	1 (2%)	9 (25%)	26 (9%)
Doctorate	0 (0%)	1 (4%)	2 (1%)	0 (0%)	0 (0%)	3 (1%)
MPH	2 (6%)	2 (7%)	2 (1%)	1 (2%)	0 (0%)	7 (3%)
Others	7 (20%)	2 (7%)	2 (1%)	7 (17%)	21 (58%)	39 (14%)
Years worked in PEC						
0–5	7 (21%)	17 (61%)	87 (65%)	30 (73%)	13 (36%)	154 (56%)
6–10	16 (47%)	6 (21%)	18 (13%)	8 (20%)	7 (19%)	55 (20%)
11–15	4 (12%)	2 (7%)	13 (10%)	1 (2%)	11 (31%)	31 (11%)
16+	7 (21%)	3 (11%)	16 (12%)	2 (5%)	5 (14%)	33 (12%)
Role*						
Physician	7 (20%)	18 (64%)	82 (61%)	10 (24%)	9 (25%)	126 (46%)
Prehospital professional	24 (69%)	10 (36%)	16 (12%)	13 (32%)	18 (50%)	81 (30%)
Medical Director	1 (3%)	9 (32%)	18 (13%)	8 (20%)	6 (17%)	42 (15%)
Hospital Nurse	3 (9%)	0 (0%)	20 (15%)	14 (34%)	0 (0%)	37 (14%)
Prehospital emergency care administrator (nonmedical)	5 (14%)	0 (0%)	3 (2%)	0 (0%)	1 (3%)	9 (3%)
Dispatcher/telecommunicator	8 (23%)	0 (0%)	7 (5%)	0 (0%)	6 (17%)	21 (8%)
Other	5 (14%)	1 (4%)	10 (7%)	4 (10%)	8 (22%)	28 (10%)
Area of work						
Rural	3 (9%)	4 (14%)	94 (71%)	4 (10%)	1 (3%)	106 (39%)
Urban	18 (51%)	11 (39%)	27 (20%)	13 (32%)	13 (36%)	82 (30%)
Mixed (rural and urban)	14 (40%)	13 (46%)	11 (8%)	24 (59%)	22 (61%)	84 (31%)

* Participants could select more than one response; percentages may total over 100%.

*EMT*, emergency medical technician; *MD/MBBS/DO*, doctor of medicine, Bachelor of Medicine, Bachelor of Surgery; doctor of osteopathic medicine; *MPH*, master of public health; *RN/MSN*, registered nurse; master of science in nursing; *PEC*, prehospital emergency care; *PECSET*, Prehospital Emergency Care System Evaluation Tool.

**Table 4:** Top-ranked Prehospital Emergency Care System Evaluation Tool elements for implementation priority by country. This table identifies the two specific elements (eg, layperson education, dispatch-assisted instructions, or staff wellness) that stakeholders in each country prioritized for implementation within the next six months in a study of the acceptability of the Prehospital Emergency Care System Evaluation Tool among Southeast Asian stakeholders.

Element	Countries Prioritizing that Element
Dispatch-assisted instructions	The Philippines, Malaysia, Vietnam
Essential patient care record documentation and medical record management	Thailand*
Layperson public awareness and education	The Philippines, Indonesia, Malaysia, Thailand*, Vietnam
Prehospital staff wellness program	Thailand*
Standardized training programs for community first responders, dispatchers, and prehospital personnel	Indonesia, Thailand*

* Thailand had three elements tied for its second most popular element.
