# Supplementary material for: Acceptability of a Novel Prehospital Emergency Care System Evaluation Tool Among Southeast Asian Stakeholders: A Cross-Sectional Survey
Source: West J Emerg Med. 2026 Nov 1;27(6):1642–51. doi: 10.5811/westjem.61956 (PMC13761771; doi:10.5811/westjem.61956)
Supplement: Supplementary file 1 [file wjem-27-1642-s001.pdf]

PEC Systems Evaluation Tool – Basic Core

Name (optional)

____

Emergency Medical Services System name

____

Select all roles below that apply to your current position:

- ☐ Prehospital emergency care medical director
- ☐ Emergency department medical director
- ☐ Emergency medicine physician
- ☐ Prehospital emergency medicine physician
- ☐ Physician
- ☐ Paramedic
- ☐ Emergency Medical Technician
- ☐ Hospital Nurse
- ☐ Prehospital nurse
- ☐ Prehospital emergency care administrator (non-medical)
- ☐ Dispatcher/telecommunicator
- ☐ Other(s):____

Core Elements

Please indicate if the following element is implemented in your system?

Please click below link to download the list of definitions: Final list of elements

	Not Implemented	Partially Implemented	Fully Implemented	Unsure
Availability of a universal access number for all medical emergencies	○	○	○	○
Dispatch-assisted instructions (eg. first aid, CPR instructions)	○	○	○	○
Essential patient care record documentation and medical record management	○	○	○	○
Layperson public awareness and education programs	○	○	○	○
Medical direction or control and access to protocols	○	○	○	○
Medical records management, dataset collection, and registry	○	○	○	○
Minimum national or regional standard requirements for an ambulance	○	○	○	○
Operational metrics for prehospital emergency care services	○	○	○	○
Operational safety protocols	○	○	○	○

Protocols for screening, triage and appropriate destination	○	○	○	○
Quality assurance or quality improvement system in place	○	○	○	○
Standardised training programs for community first responders (CFR), dispatchers, and prehospital personnel (paramedics, etc)	○	○	○	○
