# Supplementary material for: Acceptability of a Novel Prehospital Emergency Care System Evaluation Tool Among Southeast Asian Stakeholders: A Cross-Sectional Survey
Source: West J Emerg Med. 2026 Nov 1;27(6):1642–51. doi: 10.5811/westjem.61956 (PMC13761771; doi:10.5811/westjem.61956)
Supplement: Supplementary file 2 [file wjem-27-1642-s002.pdf]

PEC System Evaluation Tool (PECSET)

Personal and professional background The following questions ask about your professional and personal background, training, and the organization you work for.

Emergency Medicine System name: ______________________________

Country of work

- ◯ Philippines
- ◯ Indonesia
- ◯ Malaysia
- ◯ Thailand
- ◯ Vietnam
- ◯ Other: _____

City or region of work ______________________________

Please select the response that most accurately matches the area where you work.	◯ Rural<br>◯ Urban<br>◯ Mixed (rural and urban)
What are your credentials? (please select all that apply)	☐ MD - Medical Doctor<br>☐ MBBS - Bachelor of Medicine, Bachelor of Surgery<br>☐ DO - Doctor of Osteopathic Medicine<br>☐ BSN - Bachelor of Science in Nursing<br>☐ RN - Registered Nurse<br>☐ MSN - Master of Science in Nursing<br>☐ EMT - Emergency Medical Technician<br>☐ Doctorate<br>☐ MPH - Master of Public Health<br>☐ Other(s): ______
How many years have you been working in prehospital emergency care?	◯ 0-5 years<br>◯ 6-10 years<br>◯ 11-15 years<br>◯ 16+ years
Select all roles below that apply to your current position:	☐ Prehospital emergency care medical director<br>☐ Emergency department medical director<br>☐ Emergency medicine physician<br>☐ Prehospital emergency medicine physician<br>☐ Physician<br>☐ Paramedic<br>☐ Emergency Medical Technician<br>☐ Hospital Nurse<br>☐ Prehospital nurse<br>☐ Prehospital emergency care administrator (non-medical)<br>☐ Dispatcher/telecommunicator<br>☐ Other(s): _____

Plans to use and/or share the tool The following questions ask about your plans to use and/or share the Prehospital Emergency Care System Evaluation Tool (PECSET).

Do you plan to use the tool as part of your work?	◯ Yes<br>◯ No
Please explain how you might use it:	________________________________
Please explain why not:	________________________________

What two elements of the tool (e.g., responder training, dispatch instructions) will you focus on over the next 6 months?

- Select the elements in the dropdowns below.
- Download the pdf below for a list of the elements and their definitions.

[Attachment: "Final list of elements_20230424.pdf"]

Element 1:

- ◯ Ability to receive feedback from patients utilising the ambulance services
- ◯ Air transport infrastructure
- ◯ Availability of a universal access number for all medical emergencies
- ◯ Dedicated medical teams in hospital to receive pre-hospital patients
- ◯ Dispatch-assisted instructions (eg. first aid, CPR instructions)
- ◯ Essential patient care record documentation and medical record management
- ◯ Feedback mechanism for pre-hospital staff
- ◯ Formal interfacility transfer system with protocols and monitoring/quality assurance
- ◯ Formal recognition of prehospital care within a governmental structure
- ◯ Infrastructure for the development of teleconsult or teleradiology service
- ◯ Layperson public awareness and education programs
- ◯ Mechanism to obtain hospital or community data on patient outcomes
- ◯ Medical direction or control and access to protocols
- ◯ Medical records management, dataset collection, and registry
- ◯ Minimum national or regional standard requirements for an ambulance
- ◯ Networks with representatives spanning across the chain of survival for each time sensitive clinical domain
- ◯ Operational metrics for prehospital emergency care services
- ◯ Operational safety protocols
- ◯ Pre-hospital notification and/or activation system
- ◯ Prehospital staff wellness program
- ◯ Protocols for Infection control/ disease outbreak
- ◯ Protocols for mass casualty incidents and disaster management
- ◯ Protocols for prehospital surge capacity situations
- ◯ Protocols for screening, triage and appropriate destination
- ◯ Protocols to respond to surge in the ED
- ◯ Providing appropriate care bundle on the ambulance
- ◯ Public AED availability and usage
- ◯ Quality assurance or quality improvement system in place
- ◯ Ratio of numbers of pre-hospital ambulances and staff to the population being served
- ◯ Scope of practice defined at a national or regional level
- ◯ Standardised training programs for community first responders, dispatchers (CFR), and prehospital personnel (paramedics, etc)
- ◯ Standardized pre-hospital clinical assessment/ scoring tool

Element 2:

- ◯ Ability to receive feedback from patients utilising the ambulance services
- ◯ Air transport infrastructure
- ◯ Availability of a universal access number for all medical emergencies
- ◯ Dedicated medical teams in hospital to receive pre-hospital patients
- ◯ Dispatch-assisted instructions (eg. first aid, CPR instructions)
- ◯ Essential patient care record documentation and medical record management
- ◯ Feedback mechanism for pre-hospital staff
- ◯ Formal interfacility transfer system with protocols and monitoring/quality assurance
- ◯ Formal recognition of prehospital care within a governmental structure
- ◯ Infrastructure for the development of teleconsult or teleradiology service
- ◯ Layperson public awareness and education programs
- ◯ Mechanism to obtain hospital or community data on patient outcomes
- ◯ Medical direction or control and access to protocols
- ◯ Medical records management, dataset collection, and registry
- ◯ Minimum national or regional standard requirements for an ambulance
- ◯ Networks with representatives spanning across the chain of survival for each time sensitive clinical domain
- ◯ Operational metrics for prehospital emergency care services
- ◯ Operational safety protocols
- ◯ Pre-hospital notification and/or activation system
- ◯ Prehospital staff wellness program
- ◯ Protocols for Infection control/ disease outbreak
- ◯ Protocols for mass casualty incidents and disaster management
- ◯ Protocols for prehospital surge capacity situations
- ◯ Protocols for screening, triage and appropriate destination
- ◯ Protocols to respond to surge in the ED
- ◯ Providing appropriate care bundle on the ambulance
- ◯ Public AED availability and usage
- ◯ Quality assurance or quality improvement system in place
- ◯ Ratio of numbers of pre-hospital ambulances and staff to the population being served
- ◯ Scope of practice defined at a national or regional level
- ◯ Standardised training programs for community first responders, dispatchers (CFR), and prehospital personnel (paramedics, etc)
- ◯ Standardized pre-hospital clinical assessment/ scoring tool

Please use the scale below to respond with how likely you are to use and/or share the basic PECSET?

	I - Extremely unlikely	2 - Somewhat unlikely	3- Likely	4 - Extremely likely
How likely are you to use the elements from the basic PECSET for quality improvement?	○	○	○	○

Why are you unlikely to use the elements from the basic PECSET for quality improvement?

____

	I - Extremely unlikely	2 - Somewhat unlikely	3- Likely	4 - Extremely likely
How likely are you to share the basic PECSET with other medical directors or EMS Systems?	○	○	○	○

Why are you unlikely to share the basic PECSET with other medical directors or EMS Systems?

____

Please use the scale below to respond with how confident you are to apply the basic PECSET tool.

	I - Not at all confident	2 - Somewhat confident	3 - Very confident	4 - Extremely confident
How confident are you that you can apply the tool to your area of work?	○	○	○	○

Why are you not confident that you can apply the tool to your area of work?

____

	I - Not at all confident	2 - Somewhat confident	3 - Very confident	4 - Extremely confident
How confident are you that the tool will improve care and outcomes in your city?	○	○	○	○

Why are you not confident that the tool will improve care and outcomes in your city?

____
